# Syphilis—Signs, Symptoms, and Treatment

**Published:** 1887-03

**Authors:** J. N. Martin


(CHAPTER II.)

### Syphilis—Signs, Symptoms, and Treatment.

BY J. N. MARTIN, M. D.

(Lecture on Oral Surgery and Pathology, Dental Department, University of Michigan.)

Syphilis always begins with superficial lesions or manifestations, and step by step progresses to deeper and more serious ones. It runs a regular course from bad to worse, if not modified by treatment.

After exposure to syphilitic virus, some time elapses before there are any signs of this dreadful disease; the patient little suspects that he is inoculated with one of the worst diseases that flesh is heir to.

### PERIOD OF INCUBATION.

Usually about one month elapses between time of exposure and appearance of first sore or lesion. This is called the *period of incubation.* This period may be as short as ten or twelve days, and as long as one hundred days. The first manifestation is called by some hard chancre, by others, first lesion or initial lesion.

### FIRST LESION OR SORE.

This always occurs at seat of inoculation, or where virus entered system, and after distinct period of incubation. Its characteristics are:

1. Usually single sore or ulcer, situated on an indurated base, somewhat elastic generally.
2. Edges of ulcer not undermined but sloping.
3. Secretions very limited and watery.
4. Glands near become enlarged and hard, but do not, as a rule, inflame and suppurate.
5. Has tendency to heal rapidly, disappearing in a few days generally without treatment. Usually there is a period of freedom of from one to three months before disease takes another step. This period is called by some *second period of incubation.*

There are generally some premonitory symptoms for a few days, such as fever, headache, pain in muscles and bones (worse at night), and general feeling of exhaustion, and then an eruption appears, which rapidly spreads over the whole body and limbs ; eruption pinkish red color, gradually changing to reddish brown, or copper color, fading away in about a month. The disease becoming more general now, the *glands all over the body enlarge* and become hard. This is an important diagnostic point.

About the time this eruption disappears (little before or little after) another eruption of papular nature is ushered in ; elevated eruption, red color, changing to brownish red, or copper color, scaly at last. This stage remains two or three months. During this stage hair may drop out, but returns in short time usually, as hair follicles are not destroyed.

PUSTULES.

Following the papular stage from six to twenty months, is the stage of pustules, which begin deep down in the true skin, yellow from pus contained within ; these pustules may occur in any part of the body. Often great crusts or scabs form from exudations from the pustules, and when crusts are removed ulcers are exposed. These pustules remain three to five months, and are decidedly more filthy than the papules: patient not only loathsome to others, but to himself, for in some cases large areas are covered by this filthy exudation. The venereal wards of the hospitals of New York City and other large cities show hundreds of cases in this stage. These pustules are confined to the skin, or mucous membrane.

GUMMATA.

The disease progresses, and in from two to six years the deeper tissues, as well as the skin, are infiltrated ; hard masses form in different parts of the body, more often the limbs, which may be well defined, or blend with surrounding tissues ; these hard masses from infiltration are called *gummata.* These masses are prone to degenerate, or break down, and large, filthy, and ragged ulcers result, from which the secretions are abundant, thus making the sufferer more repulsive than before.

During this stage the patient may be literally bored full of holes, bones eaten away, teeth lost. This is the stage when severe periostitis, osteitis, caries, necrosis, and abscess occur, and if hair drop out during this stage it does not return, for hair follicles are destroyed.

This is called by some the *tertiary* stage; the stage when nose, tongue, eyesight, and hearing may be destroyed; in fact, gummata may form in any organ and destroy it, or impair its functions. These deep, ragged sores, are the *true syphilitic ulcers.* By watching the progress, it is easy to see how this disease, step by step, grows worse, the lesions extending deeper and deeper into the tissues; beginning as slight superficial eruption and ending in terrible destruction of deep structures and important organs, against which no organ is insured.

I have confined myself principally to the signs and symptoms in the skin thus far, and purposely.

MUCOUS MEMBRANES.

Corresponding to the syphilitic manifestations in the skin, in time, are similar ones, but more persistent in mucous membranes, which are a greater source of annoyance and more prone to repeat themselves than those of the skin. As on the skin, they begin as superficial lesions, and grow deeper and more destructive.

During stage of skin eruption, mucous membrane of throat, cheeks, lips, and tongue inflame and are covered with eruption; patient complains of sore throat, which is persistent. Ordinary remedies fail to relieve it. The spotted appearance disappears and returns, perhaps, several times; each time deeper tissues are involved.

MUCOUS PATCHES OR ULCERS.

Later mucous membranes are infiltrated, and over these thickened portions slight erosions result; small patches, white and glistening in appearance, senstive to touch, heat and cold; they look some like fever sores, but do not blister, and do have hard and somewhat elastic base. These patches appear to be covered with whitish film, but the white is below surface, and cannot be removed, as in aphthare. These are not, as yet, true ulcers. At this time there may be some pustules in the mouth and throat.

*The proneness of these patches to return is of importance in diagnosis.*

SECOND STAGE OF SO-CALLED MUCOUS ULCERS

In time these hard masses from infiltration break down, and true ulcers result; more often found in throat, but may occur anywhere on mucous membrane. Ulcer is ragged, uneven, and covered with debris from broken down tissue. During first stage there were small white, glistening patches, with hard bases; now ragged ulcers, with destruction of tissue. These real ulcers not so sensitive to heat and cold as the first, but are aggravated more by movements of tongue, lips, and during mastication.

These hard ulcers are gummata, and when they form deep in throat may impair vocal organs. In the mucous membrane they are more persistent. These masses, when in soft movable structures, give little, if any pain, unless irritated. They are not so distinctly outlined as when in the skin.

Sometimes these gummata disappear without breaking down, but soon others come, and the more often repeated, the more prone to break down and form deep ragged ulcers. When these ulcers form in hard parts, as septum of nose, hard palate, etc., serious deformities may result.

Gummata often causes paralysis of certain sets of muscles, by injury to nerves supplying the muscles.

GENERAL SIGNS AND SYMPTOMS.

Effects on general constitution in earlier stages are not marked; some fever, etc., as noted before, but in later stages there is plain evidence that some terrible disease is preying on the system.

I have given the signs and symptoms of syphilis when allowed to take its course, not modified by treatment. It is well known that treatment will modify the course of the disease, but unless treatment is regular, proper, and persistent, the disease progresses from bad to worse, although the symptoms are somewhat modified.

The effects of hereditary syphilis on the teeth are thought by some to be of great importance. Hutchinson, of London, and others, claim that when upper incisors are notched and teeth peg-like, that it is unmistakable evidence of syphilis, while other good authorities claim that these conditions are also produced by other diseases. Acquired syphilis may, or may not, affect the teeth directly.

Hereditary syphilis usually shows itself within a year after birth; child may be born with signs and symptoms, or may be born without them, and within a few days, weeks, or months, show marked evidence of this dreadful disease.

The hereditary side of syphilis is a large field, and requires a separate chapter.

### DIAGNOSIS.

Diagnosis is made from signs and symptoms, and is of the utmost importance, for patient should not suffer the long course of treatment necessary to cure syphilis unless he is afflicted with syphilis.

### DIFFERENTIAL POINTS.

First differentiate from epithelial cancer for which it might be mistaken on lips or tongue.

### SYPHILIS.

Pain less and not sharp; before forty years of age, as a rule.

Glands enlarge early; little odor.

Frequently more than one hard mass or ulcer.

Other signs and symptoms, as eruptions, pustules, etc., not found in epithelioma.

### EPITHELIOMA.

More pain, and generally sharp.

After forty years of age, generally.

Glands enlarge later.

Considerable odor generally.

Usually but one hardness.

### SYPHILIS.

Comes slowly. Not easily recognized at first.

Erosion not covered by curdy mass.

Real ulcer; ragged edges.

White below the surface in the erosions.

Not bordered by red areola generally.

Base hard or indurated; persistent.

Other signs of syphilis.

### APHTHOUS ULCERS.

Come suddenly, after slight inflammation, and easily recognized. Ulcers generally round or oval; covered by white substance resembling cheese curd.

Ulcer bordered by red areola of inflammation.

Base not hard or indurated.

Soon disappears with or without treatment.

No other signs of syphilis.

### TREATMENT.

The treatment of syphilis is not of so much importance to the dental profession as a thorough knowledge of those signs and symptoms that enable dentists to detect the disease in order that they may shun the dangers. However, I think a few words on treatment of syphilis may not be out of place in a dental journal.

In the treatment of syphilis, it should always be remembered that there is a patient—a human being—behind the disease, and that the best remedies in the treatment of syphilis are two-edged swords, and will cut the wrong way as well as the right.

The object is to get rid of the disease and leave the patient. He who succeeds best in fighting this enemy is the one who can skillfully use these remedies to slay the disease, and at the same time keep up the powers of his patient. This means much in treating syphilis.

. If in any branch of medicine we can truthfully say that we have specific remedies, it is in the use of those agents for treating syphilis which have proven most reliable, viz., mercurials, iodides, and tonics.

The patches, or ulcers, about the mouth and throat, may be touched with stick of nitrate of silver, or solution of nitrate of silver, or other astringents, and thus make patient more comfortable, but not cure him.

### CURATIVE TREATMENT.

When certain that patient has syphilis, use the sheet anchors —mercurials in early stages, and iodides in later stages; tonics with both, to fortify the system against the deleterious effects of remedies as well as disease.

Correct all derangements as far as possible, and thus keep general system in as good condition as possible.

Tonics are of the utmost importance, as these alteratives have a tendency to derange digestion. Alteratives generally produce less disturbance when taken after meals, and with tonics.

Some patients do better on "mixed treatment," a combination of mercurials and iodides.

The treatment should be systematic, thorough, and continued from one to two years, and, by the skillful use of these remedies, in most cases the bad symptoms disappear as by magic, and patient may be cured.

Patient should be carefully watched, avoid salivation, meet all derangements, and, above all, while it is often necessary to use large doses, do not injure the patient more by the remedies than he would be injured by the disease.

